# Staphylococcal toxic shock syndrome in a lactating mother with breast abscess: A case report

**DOI:** 10.1016/j.amsu.2020.07.027

**Published:** 2020-07-22

**Authors:** Kamal Pandit, Sushil Khanal, Prabhat Adhikari, Samaj Adhikari, Subhash Prasad Acharya

**Affiliations:** a Department of Critical Care, Grande International Hospital, Kathmandu, Nepal; b Department of Infectious Disease, Grande International Hospital, Kathmandu, Nepal; c Institute of Medicine, Maharajgunj, Kathmandu, Nepal; d Department of Anaesthesiology, Institute of Medicine, Maharajgunj, Kathmandu, Nepal

**Keywords:** Staphylococcal toxic shock syndrome (TSS), Breast abscess, MRSA, Incision and drainage, Lactation

## Abstract

**Introduction:**

The highest risk for Staphylococcal Toxic Shock Syndrome are female patients with pre-existing Staphylococcal vaginal colonization who frequently use contraceptive sponges, diaphragms or tampons. In addition patients with burns, soft tissue injures, retained nasal packing, post-abortion, post-surgical, post intrauterine device placement and abscess formation are also at high risk.

**Case presentation:**

A 19 years old female complaint of high fever with altered level of consciousness. She also had history of nausea, vomiting, diarrhea and pain on her left breast for 5 days. She developed desquamation on her palms and soles on the day three of her admission to ICU. Ultrasonography of her left breast showed 2*2*1 cm abscess collection and the culture report from breast abscess showed *Staphylococcus aureus*, sensitive to clindamycin, vancomycin and resistant to methicillin. She showed clinical improvement after commencing vancomycin and clindamycin as per culture sensitivity report of breast abscess.

**Discussion:**

Toxic shock syndrome secondary to breast abscess in adult is infrequently reported. The diagnosis of Toxic shock syndrome is made by the Centers for Disease Control and Prevention (CDC) definition. Antibiotics for treatment of this condition should include a penicillinase-resistant penicillin, cephalosporin, or vancomycin (in methicillin-resistant *S. aureus* prevalent areas) in combination with either clindamycin or linezolid.

**Conclusion:**

Treatment for breast abscess warrants incision and drainage as important as antibiotics with anti-toxin. Focused history, physical examination, and laboratory investigations are crucial for the diagnosis and management of this condition.

## Highlights

• The average annual incidence of TSS among adults is low and MRSA isolation among those cases is even more rare.
• TSS secondary to breast abscess in adult is infrequently reported.
• Treatment for breast abscess warrants I &D as important as antibiotics with anti-toxin.
• TSS is a potentially deadly condition and it requires prompt recognition and management.

## 1 Introduction

*Staphylococcus aureus* causes toxic shock syndrome (TSS) by staphylococcus toxin, especially TSS toxin-1 (TSST-1), staphylococcal enterotoxin B and enterotoxins types A, C, D, E and H [1]. These act as super antigens, activating the T lymphocytes with massive release of proinflammatory cytokines responsible for fever, rash, septic shock, and multiple organ failures [[1], [2], [3], [4]]. Staphylococcal TSS presents with varied clinical pictures which depend on case severity and immune status of the patient. The average annual incidence of TSS among adults is 11 per 100,000 and Methicillin-resistant *Staphylococcus aureus* (MRSA) isolation among those cases is even more rare [5,6]. Death rate can vary up to 30–80% [[5], [6], [7]] depending upon patient's comorbidities and disease course. The infection site being remote to the skin involvement with no bullae formation and negative nikolsky's sign differentiate staphylococcal TSS with Staphylococcal scalded skin syndrome (SSSS) which is more common in children and presents as bullae formation with positive nikolsky's sign [5].

Here we present a rare case of Staphylococcal TSS in a lactating mother with breast abscess.

### 1.1 Case summary

A 19 years old female was brought to the emergency department with a chief complaint of fever (recorded up to 102**°**F at home) with altered level of consciousness. She also had history of nausea, vomiting and diarrhea for 5 days. The patient further complained of pain on her left breast. Her urine output was less than usual. She had a history of normal vaginal delivery 8 weeks ago and was breastfeeding her child. Otherwise she did not have significant past medical and surgical history. She was nonsmoker and non-diabetic. She was not taking any immunosuppressive agents. On presentation to emergency department, she was drowsy and confused with Glasgow Coma Scale (GCS) of E3V4M6. Her initial blood pressure was 80/60 mm of Hg, heart rate was 98/min, respiratory rate was 20/min and temperature was 102.8**°**F. Her eyes and oral mucosa were dry and her capillary refill time was more than three seconds. Icterus was noted however, pallor, lymph node enlargements and rashes were absent.

Her abdominal exam revealed minimal tenderness in the right upper quadrant with no peritoneal signs. Examination of her breasts revealed mild tenderness on the right outer quadrant of the left breast, with signs of inflammation on the overlying skin without any obvious discharge.

Her pelvic examination did not reveal any discharge per vagina and there was no tenderness on palpation.

### 1.2 Investigations

Laboratory examinations showed white blood cells (WBC) 15,850 per mm^3^, hematocrit 36%, hemoglobin 12.1 g/dl, platelets 282,000 per mm^3^, creatinine 1.92 mg/dl, BUN (blood urea nitrogen) 35 mg/dl, glucose 82 mg/dl, pH 7.28 and lactate 1.4 mg/dl.

Other investigations showed total bilirubin (TB) 2.7 mg/dl, direct bilirubin (DB) 1.6 mg/dl, aspartate aminotransferase (AST) 90 U/L, alanine aminotransferase (ALT) 150 U/L, prothrombin time (PT) 15 seconds, INR 1.2 and APTT 38 seconds.

Chest x-ray was done and revealed normal findings. A lumbar puncture was performed and the cerebrospinal fluid (CSF) showed no abnormal cytological or biochemical changes.

Ultrasonography (USG) of her left breast showed 2*2*1 cm cystic area and there was probe tenderness. The aspirant containing pus was sent for culture sensitivity. The culture report from breast abscess showed *Staphylococcus aureus* sensitive to chloramphenicol, ciprofloxacin, levofloxacin, moxifloxacin, trimethoprim/sufamethoxazole, clindamycin, erythromycin, gentamicin, vancomycin and resistant to methicillin and piperacillin-tazobactum. The blood culture, urine culture and vaginal swab culture showed no growth. Stool microscopy was normal, and culture showed no growth.

### 1.3 Treatment, outcomes and follow ups

Resuscitation was started at emergency department with 1.5 L of fluid bolus of normal saline. Despite fluid bolus no improvement in blood pressure and tissue perfusion were noted, and vasopressors (noradrenaline and adrenaline) were started and titrated. Piperacillin-tazobactam was started empirically in emergency after sending blood culture.

**Fig. 1:**
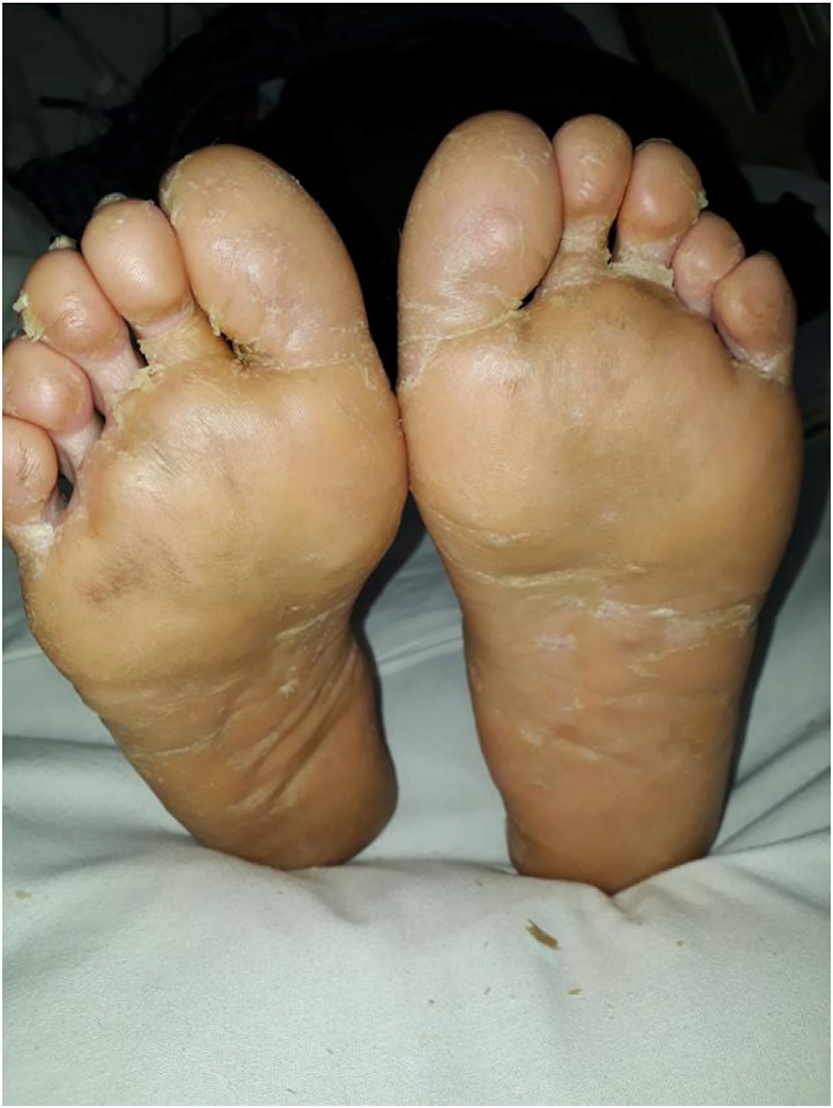
Desquamation on both soles on third day of admission.

**Fig. 2:**
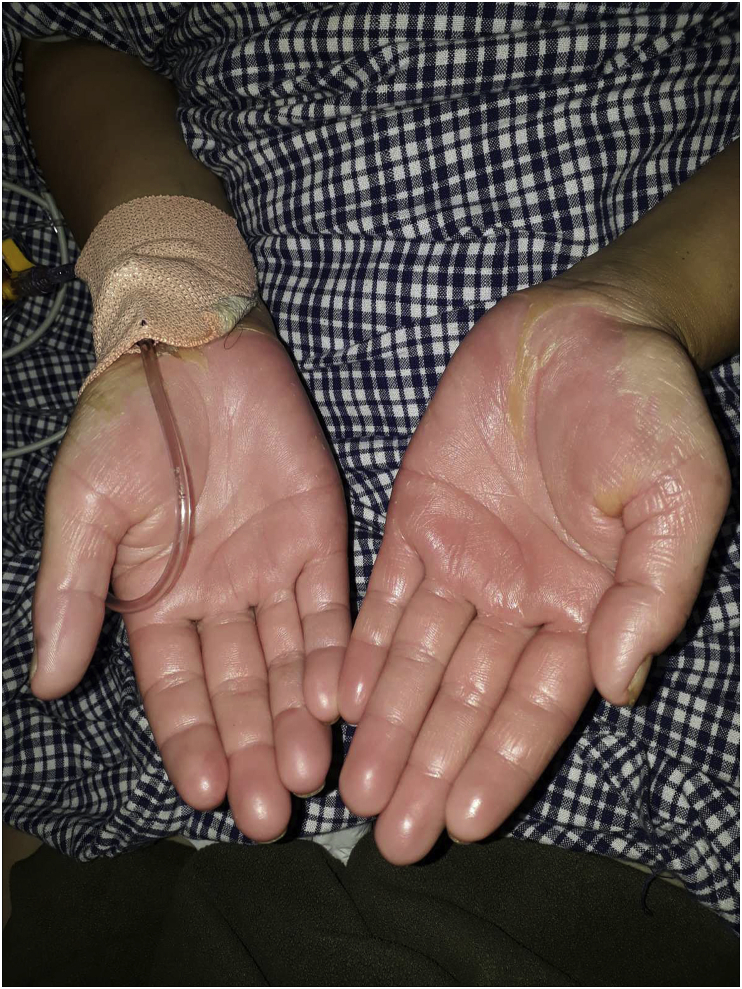
Desquamation on both palms on sixth day of admission.

**Fig. 3:**
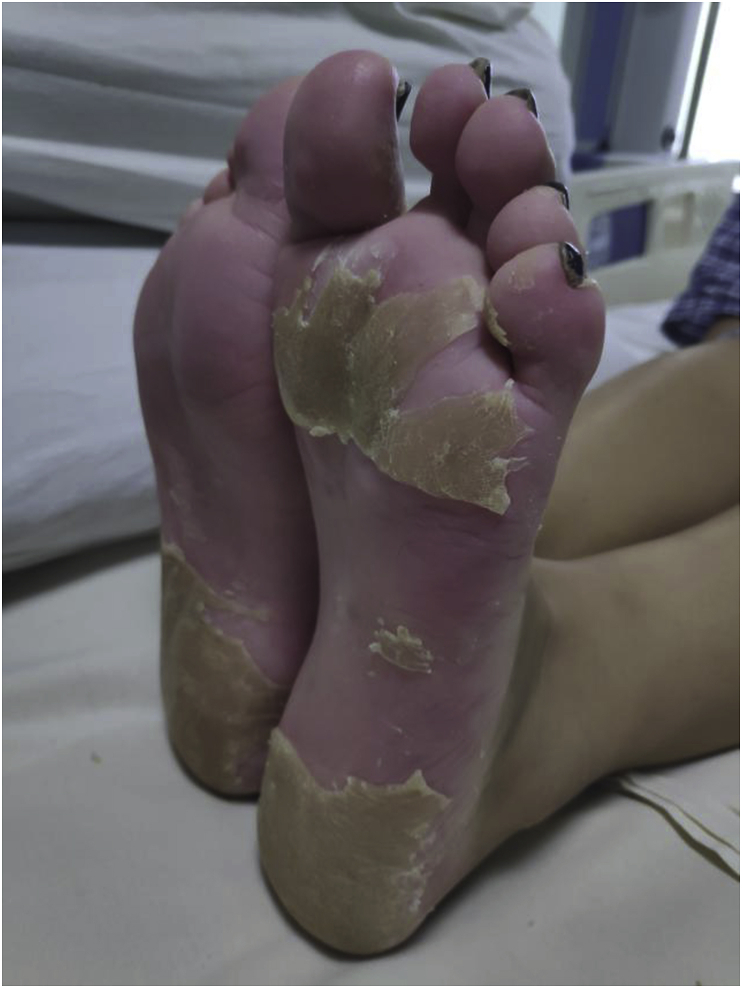
Complete desquamation on both soles on 7th of admission

The patient was shifted to intensive care unit (ICU). On the same day of her admission to ICU after confirmation of breast abscess by USG and aspiration, incision and drainage (I&D) of breast abscess was done on the bed side under local anesthesia with intravenous supplemental opioid analgesia. The surgical team was satisfied with the drainage of the abscess and there was no need to have a further exploration later on. However there was not expected improvement with I&D in initial few days. She developed desquamation on her palms and soles as shown in Fig. 1, Fig. 2, Fig. 3 from day three of her diagnosis of breast abscess by USG on ICU. She showed clinical improvement only after commencing vancomycin and clindamycin as per culture sensitivity report of her breast abscess. Additionally there was correction of renal and hepatic functions with normalization of the inflammatory markers.

The patient was discharged from the hospital two weeks after admission on oral clindamycin and on her follow up visit after a week of her discharge she was completely asymptomatic.

## 2 Discussion

According to CDC criteria [8], TSS diagnosis is supported by the following clinical and laboratory criteria:

● Fever: temperature greater than or equal to 102.0 °F (greater than or equal to 38.9 °C)
● Rash: diffuse macular erythroderma
● Desquamation: 1–2 weeks after onset of rash
● Hypotension: systolic blood pressure less than or equal to 90 mm Hg
● Multisystem involvement (three or more of the following organ systems):
  ○ Gastrointestinal: vomiting or diarrhea at onset of illness
  ○ Muscular: severe myalgia or raised creatine phosphokinase level
  ○ Mucous membrane: vaginal, oropharyngeal, or conjunctival hyperemia
  ○ Renal: raised blood urea nitrogen or creatinine
  ○ Hepatic: raised total bilirubin, alanine aminotransferase enzyme, or aspartate aminotransferase enzyme levels
  ○ Hematologic: platelets less than 100,000/mm3
  ○ Central nervous system: disorientation or alterations in consciousness without focal neurologic signs
● Identification of *Staphylococcus aureus* on culture
● Exclusion of other etiologies from cerebrospinal fluid (CSF) studies or blood cultures i.e. other bacterial infections such as streptococcal infections, rickettsia, leptospirosis and viral infections

The most common bacteria involved in the development of a breast abscess is *Staphylococcus aureus*. TSS secondary to breast abscess in adult is infrequently reported, with just one reported case to document TSS from a breast abscess in an adult female [9]. Patients with the highest risk for Staphylococcal TSS are female patients with pre-existing Staphylococcal vaginal colonization who frequently use contraceptive sponges, diaphragms or tampons and leave them within the vagina [10]. Other risk factors include patients with burns, soft tissue injures, retained nasal packing, post-abortion, post-surgical, post intrauterine device placement and abscess formation [5,6]. Streptococcal TSS is seen in cases of pharyngitis and associated with soft tissue trauma and focal infection [5,6].

This case was confirmed by considering five criteria of the CDC definition and they were the isolation of Staphylococcus from pus culture, the presence palmar-plantar desquamation (Fig. 1, Fig. 2, Fig. 3), renal and hepatic damage, and central nervous system impairment associated with vomiting and diarrhea. However, the typical diffuse macular erythroderma rashes were not present in our case. The source of infection in this case was the abscess in her left breast and the causative agent was community acquired MRSA.The infection site was remote to the skin areas displaying the desquamation. A study has shown that 20% of lactating women develop breast abscess during their post-partum period [11]. Antibiotics for treatment of this condition should include a penicillinase-resistant penicillin, cephalosporin, or vancomycin (in methicillin-resistant *S. aureus* prevalent areas) in combination with either clindamycin or linezolid [5,6]. I & D of the breast abscess along with vancomycin and clindamycin were used for treatment in this case. Treatment for breast abscess warrants I&D as important as antibiotics with anti-toxin therapy [12]. Intravenous immunoglobulin (IVIG) is sometimes administered for presumptive TSS, however, its frequency of use and efficacy is still unclear [13]. This is only the case report to document the fulminant course of Staphylococcal TSS on a lactating mother with a breast abscess.

Treatment and monitoring of this patient were undertaken by a multidisciplinary team in the ICU. TSS is a potentially deadly condition and it requires prompt recognition and management.

### 2.1 Learning points

• The average annual incidence of TSS among adults is low and MRSA isolation among those cases is even more rare.
• TSS secondary to breast abscess in adult is infrequently reported.
• Treatment for breast abscess warrants I&D as important as antibiotics with anti-toxin.
• TSS is a potentially deadly condition and it requires prompt recognition and management.

## Please state any sources of funding for your research

This study has not received any funding.

## Ethical approval

This study was conducted in accordance with ethical standard and informed written consent was taken from patient for the publication of this case report.

## Consent

Written informed consent was obtained from the patient for publication of this case report and accompanying images. A copy of the written consent is available for review by the Editor-in-Chief of this journal on request.

## Author contribution

Please specify the contribution of each author to the paper, e.g. study concept or design, data collection, data analysis or interpretation, writing the paper, others, who have contributed in other ways should be listed as contributors.

Kamal Pandit took relevant history, clinical examination, collected relevant investigations of the patient and wrote the report revised with references. And he was directly involved in patient's care during his stay in ICU.

Sushil Khanal and Prabhat Adhikari, also wrote the report and revised it with relevant references. And they were directly involved in patient's care during his stay in ICU.

Subhash Prasad Acharya provided support and mentorship for development, writing and revision of this case report. And he was directly involved in patient's care during his stay in ICU.

Samaj Adhikari worked for literature review and revision of the case report into its final version. He was not directly involved in the patient's care.

## Registration of research studies

1.Name of the registry: Not applicable.

2.Unique Identifying number or registration ID:

3.Hyperlink to your specific registration (must be publicly accessible and will be checked):

## Guarantor

Kamal Pandit.He is the first author and corresponding author for this case report.

## Provenance and peer review

Not commissioned, externally peer reviewed.

## Declaration of competing interest

There is no any conflicts of interest.
